# Modulating the Spontaneous Adsorption of Lignin Nanoparticle at Oil‐Water Interfaces

**DOI:** 10.1002/marc.202500120

**Published:** 2025-05-14

**Authors:** Danila M. de Carvalho, Maarit H. Lahtinen, Patrícia Figueiredo, Sami P. Hirvonen, Sami Hietala, Kirsi S. Mikkonen

**Affiliations:** ^1^ Department of Food and Nutrition University of Helsinki P.O. Box 66 Helsinki FI‐00014 Finland; ^2^ Department of Chemistry University of Helsinki P.O. Box 55 Helsinki FI‐00014 Finland; ^3^ Helsinki Institute of Sustainability Science (HELSUS) University of Helsinki P.O. Box 65 Helsinki FI‐00014 Finland

**Keywords:** colloidal nanoparticles, dynamic interfacial tension analysis, industrial lignin, pH adjustment, pickering emulsion

## Abstract

The performance of (nano)particles in the stabilization of Pickering emulsions depends on their physicochemical features. Therefore, synthetic particles are commonly applied in Pickering stabilization, despite the environmental issues their use raises. Recently, lignin nanoparticles (LNPs) derived from industrial side streams have been investigated as biobased alternatives to replace synthetic stabilizers. Having a well‐defined surface chemistry, monodisperse morphology, and a unique core‐shell composition, LNPs are hypothesized to show diverse functionality and adsorption capacity at the oil‐water interface that affects the long‐term Pickering emulsion stability. To gain an understanding on the effect of various colloidal parameters, *i.e*., type of LNP, type of oil‐water system, pH, LNP concentration, and ionic strength, on the adsorption LNPs at hexadecane‐water and rapeseed oil‐water interfaces, a fundamental study using dynamic interfacial tension analysis is performed. Condition optimized for Pickering stabilization is defined and applied for preparing emulsions. Findings indicated that LNPs adsorbed spontaneously at oil‐water interfaces, which is a unique trait compared to known particles’ adsorption, usually requiring the application of high forces. LNP adsorption at interfaces is affected by conditions of colloidal parameters, with increasing of pH ensuring the greatest LNP adsorption. Emulsions stabilized with LNPs at the optimized pH 8.0 remained stable after subsequent adjustment to pH 5.0.

## Graphical abstract

The adsorption of lignin nanoparticles (LNPs) at oil‐water interfaces is investigated using the dynamic interfacial tension analysis and evaluated the effect of various control parameters on LNP adsorption capacity. Results indicated that the pH increasing supported the greater LNP adsorptions. Moreover, the functionality of LNPs in the oil‐water interface achieved by pH increasing remained after pH readjustment, supporting life science applications.

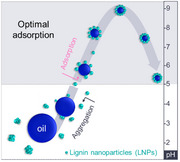

## 1 Introduction

Emulsions are dispersion systems widely used in technical and life‐science applications that, being formed by immiscible liquids such as water and oil, present high liquid‐liquid interfacial tension and physical instability. The reduction of interfacial tension and improvement of the stability of the emulsion can be achieved using stabilizers; amphiphilic surfactants for classical emulsions and colloidal (nano)particles for Pickering emulsions.^[^ ^1^ ^]^ Since the use of surfactants has been questioned nowadays regarding to their detrimental effects to the environment and health, Pickering emulsions, which can be obtained in surfactant‐free formulations, appear as relevant systems in colloidal science.^[^ ^1^, ^2^ ^]^ Pickering emulsions are suitable substitutes to classical emulsions in most applications due to similarities in their basic properties.^[^ ^1b^ ^]^ Nonetheless, the technological aspects of emulsions differ depending on the stabilizer used, i.e., whether they are particles or surfactants. For example, to achieve an efficient droplet covering during emulsion preparation, the particles that have larger sizes (10 nm to 5 µm) are required in greater amount than the surfactants that are substantially smaller (0.4–1.0 nm). Larger particles slow down the adsorption kinetics due to the relatively high energy barrier required to adsorb particles at interfaces or cause their desorption.^[^ ^3^ ^]^ Therefore, particle adsorption in Pickering emulsions is considered a non‐spontaneous phenomenon and leads to an almost irreversible particle attachment to the oil‐water interface.^[^ ^1^, ^3^, ^4^ ^]^ Consequently, Pickering emulsions are notably resistant to destabilization mechanisms as coalescence and Ostwald ripening.^[^ ^1^, ^4^, ^5^ ^]^ In addition to the size, the performance of particles is influenced by other physical‐chemical features, such as their shape, composition, charge, and wettability.^[^ ^1^, ^4^, ^6^ ^]^ Due to their well‐defined chemical structure, organic or inorganic synthetic particles have been widely explored in Pickering stabilization, despite the environmental issues that their use raises.^[^ ^2^ ^]^ In recent years, however, colloidal particles produced from industrial side streams, as the lignin nanoparticles (LNPs), started to be researched as alternatives to synthetic stabilizers to provide appropriate physico‐chemical attributes to emulsions, while increasing the sustainability, biocompatibility, safety, and cost‐efficiency of Pickering formulations.^[^ ^2^, ^7^ ^]^

LNPs are produced from lignin, an amorphous water‐insoluble aromatic biopolymer from plant's cell wall formed by phenolic monomers that are bonded together by different interlinkage patterns and functionalized at side/end positions by charged moieties.^[^ ^8^ ^]^ Currently, a large amount of industrial lignin is released from lignocellulosic‐based industries as side streams.^[^ ^8^, ^9^ ^]^ Having unique bioactivities, such as antiviral, antimicrobial, antioxidant, antimutagenic, anti‐hyperlipidemia, biodegradability, biocompatibility, UV‐blocking ability, and drug delivery,^[^ ^10^ ^]^ multifunctional systems could be obtained from lignin, if not for its complex chemical structure and water insolubility. Recently these two inconveniences were solved by transitioning industrial lignin into colloidal LNPs using simple and scalable technologies such as anti‐solvent precipitation, acidification, etc.^[^ ^9^, ^11^ ^]^ Prepared by acetone/water anti‐solvent precipitation, LNPs have a spherical, homogeneous, and monodisperse morphology, tunable particle size (90–155 nm), smooth surface, well‐defined surface properties, and great stability in a wide range of ionic strengths (10–100 mm citric acid at pH 7) and pH (4–8) conditions.^[^ ^11e^ ^]^ A peculiarity of the LNP structure is its distinct core/shell composition. The hydrophobic core contains high molecular‐weight aromatic lignin moieties while the hydrophilic shell is coated by low molecular‐weight lignin fractions and negatively charged phenolic hydroxyl and carboxylic groups.^[^ ^7^, ^11^ ^]^ Due to the concentration of negatively charged moieties at the LNP surface, LNPs are expected to respond to certain variations in the environmental conditions.^[^ ^7^, ^12^ ^]^ In application, the charged surface of LNPs induces electrostatic repulsion, a key mechanism to form aqueous colloidal nanoparticle dispersion,^[^ ^11e^ ^]^ performs emulsion interface stabilization, and prevents emulsion coalescence.^[^ ^11^, ^13^ ^]^ This tailored macromolecular arrangement unlocked a number of new opportunities for LNP application compared to industrial lignin, for example in the stabilization of oil‐in‐water Pickering emulsions,^[^ ^11^, ^14^ ^]^ preparation of agents for oil spill recovery,^[^ ^15^ ^]^ drug encapsulation,^[^ ^10c^ ^]^ and manufacture of hybrid nanocapsules with thermal energy storage capacity.^[^ ^10a^ ^]^

**Scheme 1:**
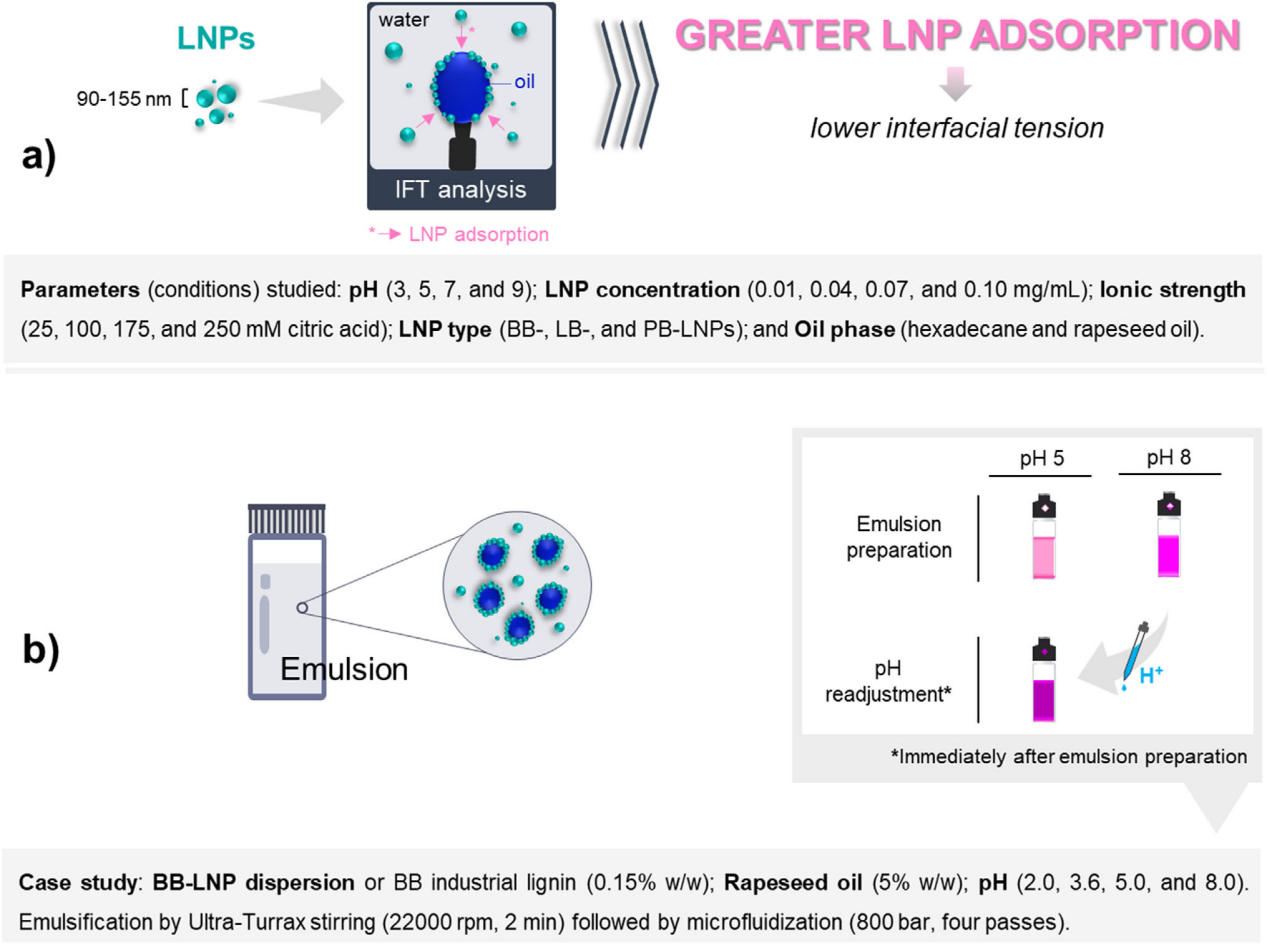
Schematic representation of LNP adsorption at oil‐water interface a) causing a decrease of interfacial tension, which was confirmed by interfacial tension (IFT) analysis and b) supporting Pickering emulsion stabilization. The methodology for readjustment of the pH of the emulsion is depicted in the gray box. Control emulsions were prepared in the absence of stabilizers.

The impact of colloidal conditions adjustment on LNP adsorption at liquid interfaces is an aspect not well understood, although crucial for advancing the application of LNPs in the development of novel and functional colloids and interfaces. Aiming to fill this knowledge gap, this study identified how the adjustment of colloidal conditions affects the LNP adsorption at the oil‐water interface and defined conditions to support the application of LNPs as stabilizers of Pickering emulsions (**Scheme** **1**). Three commercially available lignins were used to prepare LNPs: BLN hardwood birch lignin (BB), softwood kraft LignoBoost (LB), and wheat straw/Sarkanda grass Protobind^TM^ 1000 (PB). The effect of the type of LNPs and oil‐water systems, pH, LNP concentration, and ionic strength on LNP adsorption at the oil‐water interface was investigated by dynamic interfacial tension analysis, being the lower the interfacial tension value the greater the adsorption of LNPs at interfaces (Scheme 1a). Oil‐in‐water Pickering emulsions were prepared using optimized conditions for promoting LNP adsorption (Scheme 1b) and evaluated regarding their morphology and stability. Findings indicated feasible conditions to improve the adsorption of LNPs at the oil‐water interface without the requirement of an external energy input. Finally, the LNPs were identified as strongly adsorbed at the Pickering emulsion interface and resisted, to a certain extent, to the pH variation of the continuous phase, a peculiarity of colloidal LNPs to be explored in the preparation of novel systems.

## 2 Results and Discussion

### 2.1 Impact of the Adjustment of Colloidal Conditions on LNP Adsorption at Oil‐Water Interface

Target colloidal conditions, easily controlled during the preparation of oil‐water systems, were investigated regarding their impact in modulating the LNP adsorption at the interface of two systems; hexadecane‐water and rapeseed oil‐water. The decreasing of interfacial tension was interpreted as increased LNP adsorption and contrasted with reference values from control systems. The variation of pH and ionic strength in control systems caused greater response in interfacial tension to rapeseed oil due to its more complex chemical composition of rapeseed oil,^[^ ^16^ ^]^ compared to hexadecane (Figure S1 and Table S1, Supporting Information). The hexadecane, used herein as a model oil, is a pure hydrocarbon without any reactive functional groups that could respond to pH and ionic strength variation. Model systems provided a fundamental understanding on how the investigated colloidal conditions affected the surface properties and adsorption mechanisms of LNPs and provided data for the main discussion herein presented for timescale and kinetics studies.

#### 2.1.1 Timescale and Spontaneous Adsorption Kinetics

Contradicting the general assumption, LNPs spontaneously adsorbed at the oil‐water interface without using any mechanism of transport other than Brownian motion. This behavior was unexpected since individual nanoparticles in this average‐sized range (90–155 nm) are typically reported to require energy input to create a convective mechanism of transport, as turbulent flow with high shear rates, capable of promoting their adsorption at the interface.^[^ ^1^, ^3^ ^]^ Although still needing further investigation, one hypothesis would be that the hydrophilic outer layer of LNPs is soft and deformable, causing LNPs to behave more like microgels than hard particles in adsorption upon interface. Indicating that the soft outer LNP layer, by deforming at the oil‐water interface to maximize the interface coverage could allow LNP adsorption to occur in a stepwise manner, reducing the adsorption energy barrier needed.^[^ ^17^ ^]^ Additionally, outer layer deformation may contribute to LNPs being adsorbed more strongly and tightly at the oil‐water interface.^[^ ^18^ ^]^ Other soft lignin fragments, if present in the system, might also behave in a similar way. Regarding the adsorption of LNPs at the interface, different mechanisms likely govern the diffusion and the actual LNP adsorption processes. First, Brownian motion would contribute to the diffusion of LNPs from the dispersion to the interface, and then a combination of electrostatic, van der Waals, hydrophobic and capillary interactions, and interactions between LNPs would respond to their adsorption at the oil‐water interface.^[^ ^15^, ^17^ ^]^

A notable decrease in interfacial tension was noticed in the first minute of oil‐water contact. At this time point, at certain conditions, the LNP adsorption decreased the interfacial tension values by nearly 80% of those observed at the beginning of the experiments for hexadecane‐ and rapeseed oil‐water systems. This finding indicated that the LNP approaching to the interface and their consequent adsorption was relatively fast (Tables S2 and S3, Supporting Information). The longer the equilibration times the greater the decrease in interfacial tension compared to the initial values. At 10 min the interfacial tension was as low as 62.3% and 57.5% of those observed at the beginning of the experiments for hexadecane‐ and rapeseed oil‐water systems, respectively. At 60 min interfacial tension reduction indicated that 51.2 and 40.3% of the initial interfacial tension could be achieved respectively in hexadecane‐ and rapeseed oil‐water systems for certain conditions. It is worth mentioning that the greater relative variation of interfacial tension in rapeseed oil‐water systems was also influenced by the increased stability of the rapeseed oil itself in certain tested conditions, especially those more alkaline (Figure S1 and Table S1, Supporting Information).

**Figure 1:**
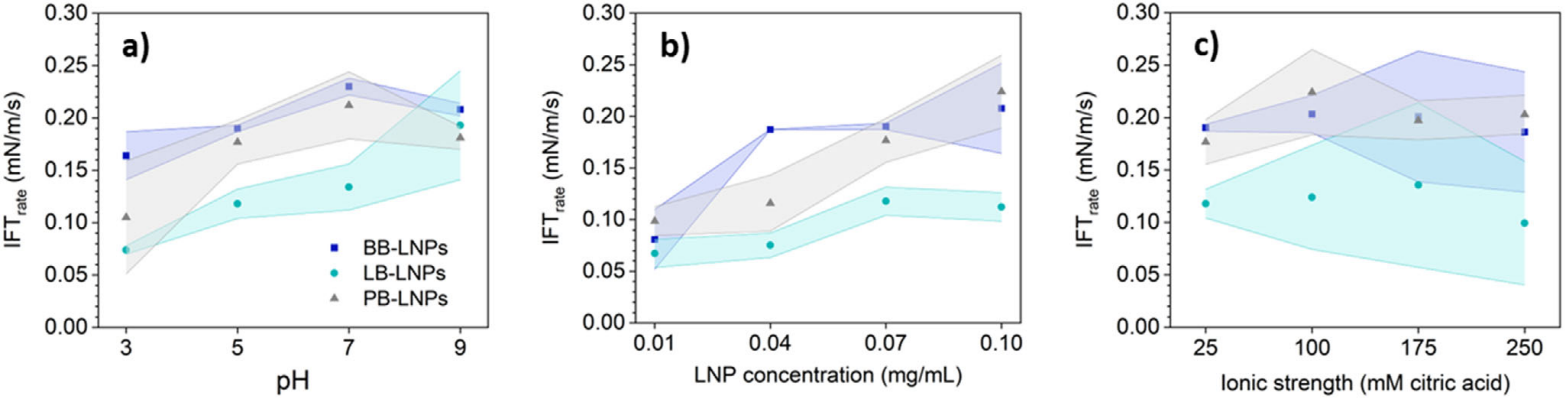
Rate of interfacial tension decrease (IFT_rate_) due to LNPs adsorption at the interface of hexadecane‐water systems as a function of a) pH, b) LNP concentration, and c) ionic strength. Values for IFT_rate_ were calculated based on the first 20 s of hexadecane‐water contact (see Experimental section for details). Unless otherwise specified, conditions for pH, LNPs concentration, and ionic strength were fixed at 5, 0.07 mg mL^−1^, and 25 mM citric acid, respectively. Colored areas represent the standard deviation.

The kinetics of the adsorption of LNPs was only studied for hexadecane‐water systems to avoid the cross‐effect of rapeseed oil contribution to the decreasing of the interfacial tension of the system. The kinetics of LNP adsorption at the beginning of the experiments (defined herein to the time range of 20 s, see Experimental section for details) was affected by the LNP type and condition applied for experiments. After initial adsorption, the rate of interfacial tension change stayed constant for all studied samples (Figure S2, Supporting Information). In general, increasing the concentration of LNPs and the pH of the systems accelerated the adsorption of LNPs at the interface (**Figure** **1**). No clear effect of variation of ionic strength was noticed. The LB‐LNPs exhibited the slower kinetics of adsorption among the LNPs investigated, something likely explained by their lower zeta potential absolute value (Table S4, Supporting Information), smaller size at neutral‐alkaline pH (90 nm) compared to their counterparts (150–155 nm), and higher susceptibility to aggregation in acidic pH.^[^ ^11e^ ^]^ This suggested that optimization of LB‐LNP dosage in hexadecane‐water systems and controlling colloidal conditions to prevent the LB‐LNP aggregation would be beneficial for promoting their use as stabilizer in oil‐water systems, something favorable including when longer equilibration times are applied (timescale study, Table S2, Supporting Information).

#### 2.1.2 LNP Adsorption at the Interface of Hexadecane‐Water Model Systems

**Figure 2:**
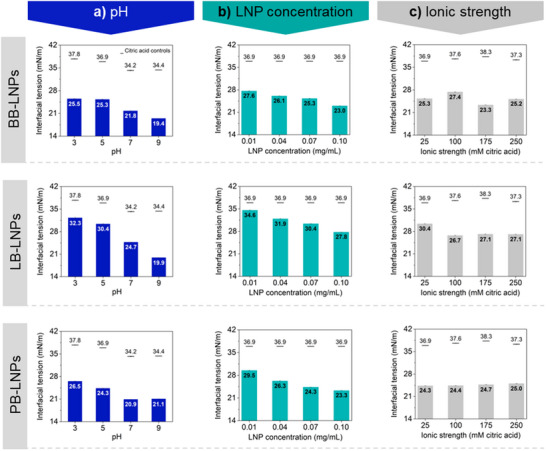
Interfacial tension of LNP dispersion‐hexadecane (bars) and citric acid controls (lines) as a function of a) pH, b) LNP concentration, and c) ionic strength at 60 min measurements. Unless otherwise specified, conditions for pH, LNPs concentration, and ionic strength were fixed at 5, 0.07 mg mL^−1^, and 25 mM citric acid, respectively. Control curves for the interfacial tension between hexadecane and the various citric acid solutions (Figure S1a, Supporting Information) and for systems stabilized by LNPs (Figure S3, Supporting Information) are presented in Supporting Information.

*Effect of pH*: The pH was the parameter affecting the LNP adsorption at interfaces most greatly (**Figure** **2**a), something explained by its capacity to modulate LNP size and charge. Regarding the LNP sizes, two different mechanisms likely occurred at the extreme pH conditions applied. At pH lower than 4, the size of the LNP increased due to their aggregation caused by the protonation of charged moieties and the consequent decrease of electrical double‐layer repulsion forces.^[^ ^11^, ^13^, ^19^ ^]^ As confirmed in a previous study, the decrease of pH to 3 caused the aggregation of LNPs due to intermolecular hydrogen bonding and protonation of carboxyl groups, which was more intense for LB‐LNPs.^[^ ^11e^ ^]^ This observation also corroborated the limited capacity of LB‐LNPs to reduce the interfacial tension at such conditions. Thus, the lowered pH contributed to reduced LNP active surface area and limited LNP capacity to establish efficient electrostatic repulsion. In turn, the deprotonation of phenolic hydroxyl groups (pKa 7–10)^[^ ^20^ ^]^ and the increase of lignin size due to pH increase can, to some extent, improve LNP surface activity. For example, at pH above 10, the structure of LNPs is expected to start to disorganize into solubilized molecules with larger sizes.^[^ ^11^, ^19^, ^21^ ^]^ The substantial decrease in the interfacial tension observed for dispersions at pH 9 suggested that certain solubilization of lignin moieties had already started at this pH. Likewise, it might be possible that some hydrophilic lignin tails from the LNP's shell were released, enabling additional interface stabilization via the amphiphilic mechanism. Regarding the functional groups, pH increase is associated to the ionization of hydroxyl and carboxylic groups,^[^ ^22^ ^]^ which improved surface activity either in LNPs or solubilized lignin in molecular form, lowering the interfacial tension values. Although the pH variation investigated herein was equidistant, the variation in interfacial tension was not constant and the greatest variation observed between pH 5 and pH 7 is likely explained by the final deprotonation of the citric acid used in the continuous phase at pH 6.4 (citric acid pKa values: pH 3.1, 4.7, and 6.4). In summary, the possibility of improving the surface properties of LNPs by simple pH adjustments clearly indicates that there is room to optimize LNP application in the preparation of stable oil‐water systems.

*Effect of LNP Type*: The various LNPs used herein were all prepared by the same approach but differed regarding the origin and extracting process (see Experimental Section, Materials and Chemicals). This led to an expected variation in the chemical structure, molar mass, and functional groups abundance of such industrial lignins and, consequently, certain differences in the properties of their correspondent LNPs, as previously published.^[^ ^11e^ ^]^ Despite such differences, fixing the LNP concentration at 0.7 mg mL^−1^ LNPs, the variation of pH affected similarly the capacity of BB‐ and PB‐LNPs to reduce the interfacial tension of hexadecane‐water systems (Figure 2a). On the other hand, the LB‐LNPs, having a smaller size and higher molar mass (90 nm, 20 kDa) than their counterparts (BB‐LNPs: 155 nm, 13 kDa; PB‐LNPs: 150 nm, 14 kDa),^[^ ^11e^ ^]^ additionally to lower absolute values of zeta potential (Table S4, Supporting Information) performed substantially less interfacial tension decrease in acidic and neutral conditions (pH 3–7). At pH 3, the inefficiency of LB‐LNPs in reducing the interfacial tension is explained by the greater aggregation process experienced by the nanoparticles.^[^ ^11e^ ^]^ However, aggregation events do not explain why LB‐LNPs were comparatively less efficient in reducing the interfacial tension of systems at pH 5 and 7, conditions under which no aggregation is expected. This deserves further investigation, but the lower absolute zeta potential of such smaller particles could indicate a low density of charged groups at the surface of LB‐LNPs.^[^ ^23^ ^]^ Therefore, the absolute amount of LB‐LNPs provided was insufficient to promote an effective oil surface packing. Only at pH 9 the values for interfacial tension observed for all LNPs in hexadecane‐water systems were within a similar and narrow range; 21.1–19.4 mN m^−1^.

*Effect of LNP Concentration*: The increase in LNP concentration from 0.01 to 0.10 mg mL^−1^ decreased the interfacial tension between hexadecane and water (Figure 2b). In absolute values, LB‐LNPs performed a lower decrease of interfacial tension, achieving only 27.8 mN m^−1^ at the more concentrated LNP dispersion, while BB‐ and PB‐LNPs achieved equivalent values in less concentrated dispersions. This suggested that in comparison to BB‐ and PB‐LNPs, a more concentrated LB‐LNP dispersion would be necessary to ensure effective oil droplet packing.

*Effect of Oil Phase in the System*: LNP adsorption is also affected by the type of oil in the system. For example, in the present study the increase in BB‐, LB‐, and PB‐LNP concentration from 0.01 to 0.10 mg mL^−1^ reduced the interfacial tension of hexadecane‐water systems by 4.6, 6.8, and 6.2 mN m^−1^ respectively. Using decane as the oil phase and only water in colloidal LNP dispersions, Lee et al ^[^ ^15^ ^]^ reported a decrease in interfacial tension of 15 mN m^−1^ for a similar increase in LNP concentration. These authors prepared the LNPs by acetone/water anti‐solvent precipitation using organosolv lignin. Therefore, the effect of LNP gradient on interfacial tension decrease should be assessed accordingly when using other oil‐water systems. Although affecting the decrease of interfacial tension less than the pH control, optimizing the LNP concentration used as a stabilizer of oil‐water systems proved to be an important aspect to be considered when using model oil.

*Effect of ionic strength*: A positive effect of the application of LNPs to the reduction of the interfacial tension between hexadecane and citric acid solutions was observed for all ionic strength conditions herein tested, however, the increased ionic strength did not compromise the adsorption of LNPs at the interface of hexadecane‐water (Figure 2c). This was a surprising result due to the known effect of increased ionic strength on promoting LNP aggregation,^[^ ^6^, ^11^, ^13^ ^]^ that herein was only noticed due to the more turbid aspect of the LNP dispersions. From a technological point of view, the minimum effect of ionic strength on LNP adsorption was positive and represented freedom when deciding a condition among those herein tested for the application of colloidal LNPs.

#### 2.1.3 LNP Adsorption at the Interface of Rapeseed Oil‐Water Systems

**Figure 3:**
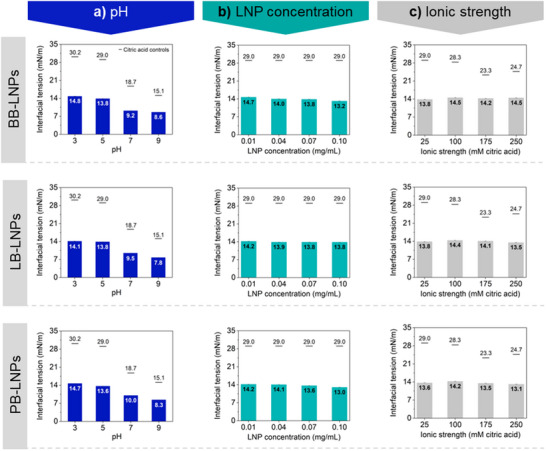
Interfacial tension of LNP dispersion‐rapeseed oil (bars) and citric acid controls (lines) as a function of a) pH, b) LNP concentration, and c) ionic strength at 60 min measurements. Unless otherwise specified, conditions for pH, LNPs concentration, and ionic strength were fixed at 5, 0.07 mg mL^−1^, and 25 mM citric acid, respectively. Control curves for the interfacial tension between hexadecane and the various citric acid solutions (Figure S1b, Supporting Information) and for systems stabilized by LNPs (Figure S4, Supporting Information) are presented in Supporting Information.

*Parameters with relevant influence on LNP adsorption – oil phase and pH*: The type of oil proved to be an important variable when studying and performing stabilization of oil‐water interfaces by LNPs, as already covered in this discussion, and reinforced with the results from rapeseed oil‐water systems. Control systems (represented as lines, Figures 2 and **3**) exhibited substantially lower interfacial tension values in rapeseed oil‐water systems than in hexadecane‐water. Furthermore, the adjustment of colloidal conditions caused a greater variation in interfacial tension values in rapeseed oil‐water systems compared to the model system. For example, taking into consideration the control systems, the increasing pH from 3 to 9 caused a much higher decrease in interfacial tension in rapeseed oil‐water systems (15.1 mN m^−1^) than that observed previously in hexadexane‐water systems (3.4 mN m^−1^) (Table S1, Supporting Information). This difference is expected due to the distinct chemical composition of these two oil types. In contrast to hexadecane, rapeseed oil contains phenolic compounds, free fatty acid, phospholipids, linoleic acid, and triglyceride, compounds potentially pH‐responsive that can be involved in interface stabilization.^[^ ^16^, ^24^ ^]^

In rapeseed oil‐water systems, the pH was also the parameter promoting the most significant decrease of interfacial tension (Figure 3a). Despite the likely aggregation of LNP at pH 3, LNP adsorption at interfaces reduced the interfacial tension by at least 15.4 mN m^−1^, compared to the controls. Although the interfacial tension at pH 9 for systems containing LNPs was only 7.3–6.5 mN m^−1^ lower than in controls, the relatively low values achieved (8.6–7.8 mN m^−1^) were highly attractive from a technological perspective. These findings pointed toward process optimization through the preparation of rapeseed oil‐water systems with increased stability due to the enhanced, and likely stronger, adsorption of LNPs at interfaces.

*Parameters with minimal influence on LNP adsorption – LNP concentration, ionic strength, and LNP type*: Fixing the pH of LNP dispersion at pH 5, the use of LNPs decreased the rapeseed oil‐water interfacial tension by at least 14.3 mN m^−1^ compared with control systems with similar performance for the various LNPs. However, the increase in concentration of LNPs from 0.01 to 0.10 mg mL^−1^ promoted only a slight decrease in the values of interfacial tension (Figure 3b). This result indicated the possibility of optimizing the preparation of rapeseed oil‐water systems with the application of lower LNP concentrations but still clearly displaying improvement in interface stabilization. Comparatively, the variation in LNP concentration from 0.01 to 0.10 mg mL^−1^ led to a more intense decrease in the interfacial tension in model systems; hexadecane‐water (4.6–6.8 mN m^−1^, Figure 2) or decane‐water (15 mN m^−1^)^[^ ^15^ ^]^ than in rapeseed oil‐water system.

Similarly, to that observed for hexadecane systems, the higher the ionic strength the more turbid the LNP dispersions became, suggesting that aggregation had occurred to some extent. Indeed, the higher decrease of interfacial tension relative to the control systems, by 15.4–13.7 mN m^−1^ in 25–100 mM citric acid dispersions compared to the interfacial tension decrease by only 11.6–9.1 mN m^−1^ achieved in 175–250 mM citric acid dispersions, corroborated with the presumable LNP aggregation caused by ionic strength increasing (Figure 3c). Although the application of LNPs substantially reduced the interfacial tension of rapeseed oil systems compared to control, only a minor effect was observed in regard to the various conditions of ionic strength (Figure 3c). To minimize the potential negative effect of ionic strength on LNP aggregation, adjusting the pH without using citric acid solutions appeared as a reasonable option for LNP application as a stabilizer in rapeseed oil‐water systems.

Unlike that observed in hexadecane‐water systems, similar interfacial tension values were observed for the three types of LNPs at different conditions of pH, LNP concentration, and ionic strength.

### 2.2 Effect of pH Adjustment on Stability of Pickering Emulsions

#### 2.2.1 Rapeseed Oil‐In‐Water Emulsions Stabilized by Colloidal BB‐LNPs

Various pH conditions, including those identified as suitable for supporting BB‐LNP adsorption at the rapeseed oil‐water interface, were applied for emulsion preparation. Emulsion preparation consisted of two sequential mixing steps. At the first step, colloidal LNP dispersion and rapeseed oil were mechanically stirred using an Ultra‐Turrax to form a coarse emulsion. Foam formation was observed for all pH conditions tested, but disappeared after stirring was stopped. The second step consisted of subjecting the coarse emulsion obtained to microfluidization by applying 800 bar pressure and a positive impact of pH adjustment on LNP adsorption at the interface was confirmed. At pH 2.0, part of the LNPs remained in the microfluidizer container and flocculated on top of the emulsion. A rapid phase separation was also noticed with the LNPs being preferentially partitioned into the oil phase, similar to that reported for the partitioning of acetylated lignin in acidic conditions.^[^ ^22^ ^]^ As the pH was increased the amount of LNPs that remained on the microfluidizer container or flocculated on top of the emulsion was reduced. Moreover, a clear improvement in emulsion homogeneity was observed for emulsion prepared at pH 5.0. Emulsions prepared at pH 8.0 were homogeneous and free from visible LNP aggregates.

**Figure 4:**
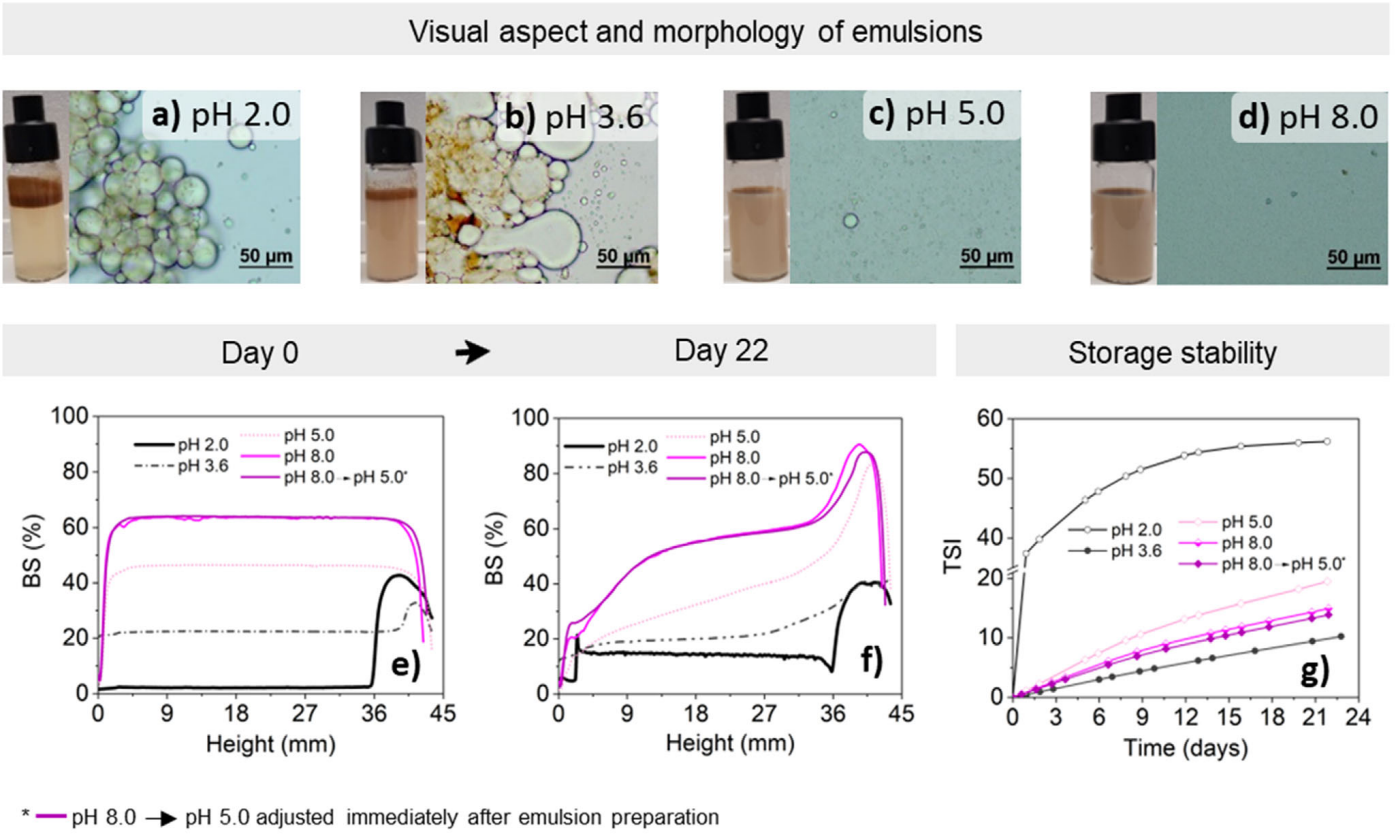
Rapeseed oil‐in‐water emulsions were prepared at a) pH 2.0, b) 3.6, c) 5.0; and d) 8.0, and the corresponding morphology of their droplets was assessed at day 0 just after emulsion preparation. Self‐assembled LNP aggregates are visible as brown particles on top of emulsions. The backscattering intensity of emulsions prepared at pH 2.0, 3.6, 5.0, and 8.0 measured e) at day 0 just after emulsion preparation and f) after storage day 22. g) Global Turbiscan Stability Index (TSI) of emulsions prepared at pH 2.0, 3.6, 5.0, and 8.0 and monitored over 22 days of storage.

Visually, it was clear that more acidic conditions at pH 2.0 and pH 3.6 were inefficient in supporting successful emulsification using LNPs as stabilizers (**Figure** **4**), consistent with the lower capacity of LNPs to adsorb at the interface and perform interfacial tension decrease at such conditions (Figure 3a). The top layer formed by self‐assembled LNP aggregates visible on top of emulsions in Figure 4a,b and increased intensity of backscattering in high height values at day 0 in Figure 4e indicated that only a small fraction of LNPs effectively adsorbed at oil droplet interface. Consequently, emulsions prepared at more acidic conditions were susceptible to droplet coalescence due to insufficient oil droplet packing by LNPs (microscopic images, Figure 4a,b). With pH increasing, the droplet size decreased along with the increase in opacity of the emulsions, indicating a more effective usage of LNPs in oil‐water interface stabilization, either by Pickering or amphiphilic mechanisms. With the adjustment of the pH of the system to 5.0 before emulsification the aggregation of LNPs on the top of the emulsion was considerably reduced, only a few particles were observed, and finer droplets were formed (Figure 4c). The adjustment to pH 8.0 before emulsification resulted in the formation of the smallest droplet sizes among the emulsions produced (Figure 4d).

After 22 days of storage, the emulsion prepared at pH 2.0 was most susceptible to droplet coalescence, evidenced by the increased intensity in backscattering at higher height (Figure 4f). In the case of emulsion prepared at pH 3.6 the main observation during storage was regarding the creaming formation on top of emulsion. The LNP flocks, already present in this emulsion since day 0, remained almost constant during storage. Although emulsions prepared at pH 5.0 and 8.0, including that readjusted to pH 5.0 after emulsification and discussed later in this section, presented some creaming formation during storage, phase separation occurred to a much lower extent than when acidic conditions were applied for emulsification.

The stability of emulsions was monitored over 22 days of storage by Turbiscan, where the lower the value for global Turbiscan Stability Index (TSI), the greater the stability (Figure 4g). As anticipated by the visual aspect of the emulsion and the morphology of the droplets, the emulsion prepared at pH 2.0 was highly unstable, exhibiting a substantial increase in TSI up to 37.4 already one day after emulsion preparation. Although the evaluation of the TSI values of the emulsion prepared at pH 3.6 seemed to be quite promising, the droplet coalescence and the flocculation of LNPs not involved in emulsification indicated that this conclusion is only partial, since the overall emulsification process was not satisfactory (Figure 4b), for which optimization was still required. Opportunely, the fundamental study carried out here indicated that increased pH is a feasible adjustment for improving the adsorption of LNPs at liquid interfaces. This strategy proved to be suitable in LNP‐applied use, resulting in a functional TSI of 19.5 for emulsion prepared at pH 5.0 after day 22 storage. Emulsion stability was even higher achieving a TSI of 15.0 when emulsion was prepared at pH 8.0 after 22 days storage. Two possible explanations for the improvement in emulsion stability due to pH increase were raised earlier in this discussion (Section 2.1.2, Effect of pH). The first relies on the observation that the more alkaline the conditions for emulsion preparation the less LNPs are self‐assembled into aggregates (Figure 4a–d), contributing to an increase the absolute amount of LNPs that effectively performed oil droplet packing. The second explanation is the potential ionization of functional groups and solubilization of BB‐LNPs caused by increasing pH leads to enhanced lignin surface activity and ensures additional stabilization via amphiphilic mechanism.^[^ ^22^ ^]^

Considering most practical applications in life science products, more neutral or slightly acidic conditions are generally desired, making pH 8, ideal for supporting LNP adsorption, unsuitable. Therefore, a further study was conducted in which the stable emulsions prepared at pH 8.0 were readjusted to pH 5.0 (gray box, Scheme 1b) and monitored during storage. Surprisingly, the backscattering and TSI results indicated that the pH readjustment of the emulsion to 5.0 not only maintained the emulsion aspect (Figure 4e,f) and stability (13.9) close to that observed for the emulsion at pH 8.0 (15.0) (Figure 4g), but also provided a feasible approach for the efficient LNP application in Pickering systems, without requiring further LNP surface modification and/or using additional stabilizer. Moreover, the readjustment of the pH to 5.0 did not affect the morphology of the emulsion interface in which the stabilizers, either in particle‐ or molecular‐form, remained strongly adsorbed after pH readjustment, as confirmed by the TSI results (Figure 4g).

Interestingly, the emulsion readjusted to pH 5.0 was more stable than that prepared initially at pH 5.0 (Figure 4f,g). A possible explanation is that the alkaline condition applied caused the release of hydrophilic lignin tails from LNP's surface, which contributed to enhancing nanoparticle wettability, steric repulsion, and preventing droplet coalescence, as observed previously for polymer‐grafted LNPs.^[^ ^14^ ^]^

#### 2.2.2 Emulsions Stabilized by BB Industrial Lignin and Control Emulsion

The eventual total or partial solubilization of BB‐LNPs at pH 8.0 could explain their improved stabilization capacity when the emulsion is prepared at alkaline conditions. However, it questions the need of using LNPs instead of the industrial lignin itself, which could also perform improved stabilization at such pH conditions. Therefore, emulsions stabilized by BB industrial lignin at pH 8.0 with and without readjustment to pH 5.0 were also investigated. Although this strategy proved to be also suitable for the direct use of molecular BB industrial lignin as stabilizers (Figure S5, Supporting Information), results confirmed that its correspondent LNPs performed superior stabilization. In the absence of stabilizers, the high interfacial tension of rapeseed oil‐water prevented these liquids from forming stable emulsions with phase separation starting already at day 2, especially for control emulsion readjusted to pH 5.0 after emulsification (Figure S6, Supporting Information).

Overall, the findings confirmed the active role of lignin species in the stabilization of oil‐water interfaces and indicated simple strategies to improve their performance as stabilizers. Furthermore, results proved the ability of LNPs to protect Pickering systems from destabilization mechanisms caused by pH variation of continuous phase from 8.0 to 5.0. This unique functionality of adsorbed LNPs has the potential to be explored in technological uses of LNPs in novel colloidal systems.

## 3 Conclusion

**Figure 5:**
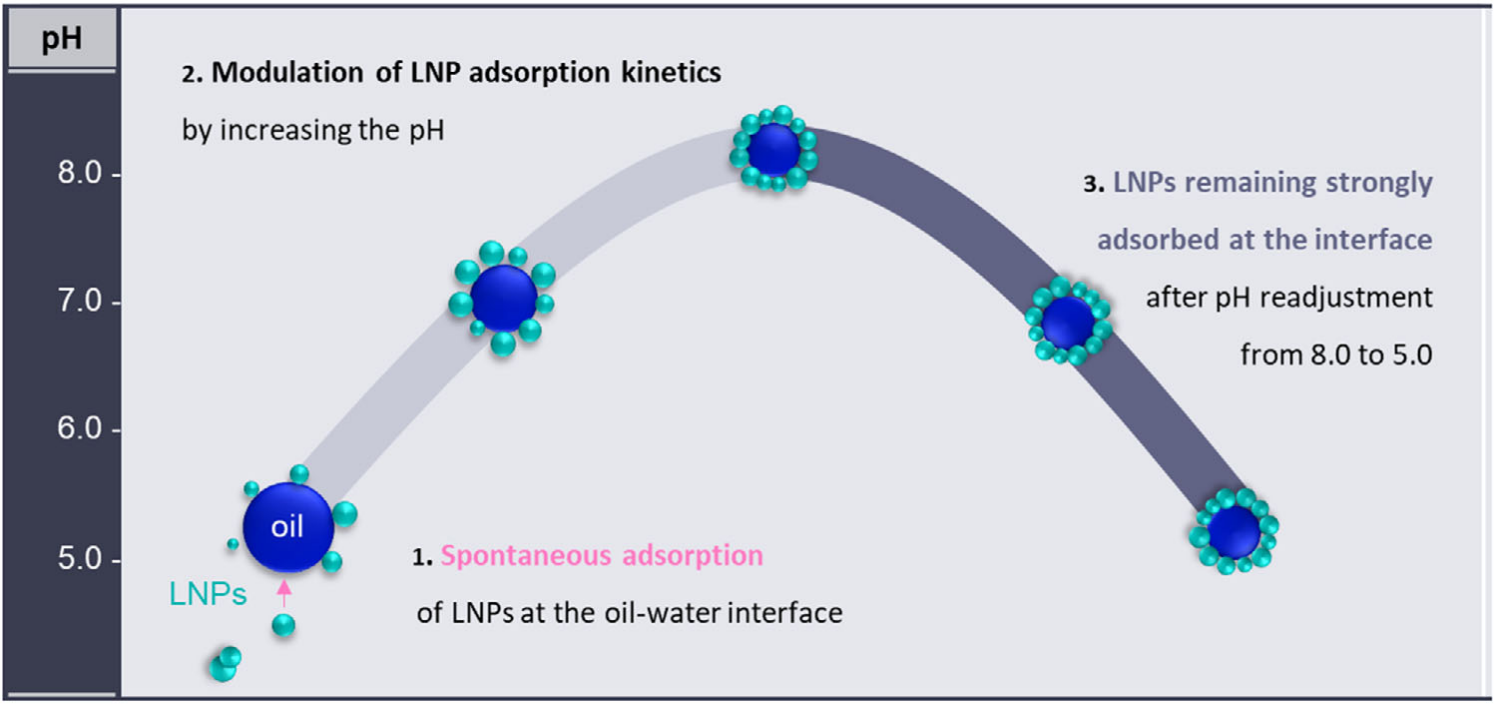
Significant findings on LNP adsorption at oil‐water interface with respect to the adjustment of pH conditions, the parameter identified to cause the greatest impact on LNP adsorption among those herein studied.

The adsorption of LNPs at hexadecane‐ and rapeseed oil‐water interfaces occurred spontaneously, revealing a unique behavior for colloidal LNPs compared to other particle stabilizers (**Figure** **5**). The adsorption of LNPs at interfaces was a time‐dependent mechanism modulated by the type and concentration of LNPs, the type of oil‐water system, pH, and ionic strength. Adjusting the pH toward alkaline conditions caused suitable modification of LNP's chemical structure, thereby supporting the greater adsorption of LNPs at interfaces in both fundamental and applied studies. Interestingly, the emulsions prepared at pH 8.0 allowed for subsequent readjustment to pH 5.0, a practical pH for life science applications, without the emulsion losing its excellent structure or stability with the LNPs remaining strongly adsorbed at the interface. Therefore, LNPs are attractive alternatives to conventional particle stabilizers in preparation of Pickering emulsion for technical and life science (safety evaluations are still pending) applications. Future studies include exploring the other bioactivities of LNPs in colloidal systems, such as antioxidant, biodegradability, biocompatibility, and drug delivery, and investigating the pH‐responsiveness of LNPs for creating novel colloids that could be used in, for example, fertilizers, water treatment, and pharmaceutics.

## 4 Experimental Section

### Material and Chemicals

LNPs prepared from three industrial lignins from different lignocellulosic sources were investigated regarding their adsorption behavior at the oil‐water interface. Hardwood birch lignin (BB), isolated through the BLN process,^[^ ^25^ ^]^ was obtained from CH Bioforce Oy (Finland). Softwood kraft LignoBoost (LB) was supplied by Stora Enso Oyj (Finland). Protobind^TM^ 1000 (PB), isolated from wheat straw/Sarkanda grass through a soda process was obtained from GreenValue SA (Switzerland). Hexadecane (> 99.0% for synthesis) was purchased from Merck (Darmstadt, Germany) and rapeseed oil was purchased from the supermarket (Apetit Ruoka Oy, Helsinki, Finland).

### LNP Preparation

LNPs were prepared by the acetone/water anti‐solvent method adapted from Farooq et al.^[^ ^26^ ^]^ and described by Figueiredo et al.^[^ ^11e^ ^]^ Briefly, 2 g of industrial lignin were solubilized in 200 mL of a mixture of acetone/water 3:1 (v/v) and stirred at room temperature for 3 h. Then, larger particles/aggregates were removed by filtration (microfiber filter with pore size of 0.7 µm, Whatman GF/F) and the lignin solution was rapidly poured into 400 mL of Milli‐Q water under vigorous stirring to form the colloidal nanoparticles dispersion. Finally, the acetone was removed by rotatory evaporation at 40–45 °C. The concentration of the LNP dispersions was determined gravimetrically after drying aliquots using an oven at 50 °C for 24 h. The resultant LNP stock dispersions were stored at 4 °C prior to use. Hydrodynamic diameter, zeta potential, and molar mass of BB‐ (155 nm, −44 mV, 13 kDa), LB‐ (90 nm, −40 mV, 20 kDa), and PB‐LNPs (150 nm, −42 mV, 14 kDa) were assessed and published previously.^[^ ^11e^ ^]^

### Interfacial Tension Study

The dynamic interaction of the various LNPs at the oil‐water interface was assessed by interfacial tension analysis, an analytical tool capable of integrating aspects from the thermodynamic state, structure of fluids interface, adsorption/desorption of stabilizers, and molecular structure of stabilizers.^[^ ^1^, ^15^, ^27^ ^]^ The analysis was performed using an optical tensiometer CAM 200 (KSV Instruments LTD, Finland). The LNP dispersions were placed in a 1 cm × 1 cm × 4.5 cm polymethyl methacrylate (PMMA) cuvette and the images were recorded after the release of a 7–9 µL pendant‐drop of the oil; hexadecane for studying technical applications or rapeseed oil for studying applications in life science. Releasing of the oil droplet was performed using a sealed syringe attached to a reverse needle with an external diameter of 0.717 mm (Figure S7, Supporting Information). After preliminary tests over 24 h to define an optimal experimental time, a period of ≈60 min was defined as a suitable time to observe the most significant variation in interfacial tension due to the variation in the tested conditions. Measurements were performed at 20 °C ± 1 °C according to the following conditions: 1000 frames in a frame interval of 20 milliseconds followed by 3600 frames in a frame interval of 1 s, totaling 4600 frames over 3620 s. Results were processed using the CAM2008 software and the interfacial tension was calculated using the Young‐Laplace fitting method (Figure S7, Supporting Information).^[^ ^15^, ^27^ ^]^

The densities of the citric acid solutions, a parameter required to calculate interfacial tension, were determined using a calibrated pipette and analytical scale with a minimum of ten repetitions. The standard deviation for densities was lower than 0.002 for all citric acid solutions. Control experiments were performed placing only citric acid solutions in the cuvette. A constant interfacial region on the pendant drop during experiments was confirmed by monitoring the droplet volume (Tables S5 and S6, Supporting Information) and area (Tables S7 and S8, Supporting Information).

Interfacial tension data at the beginning of the experiment may differ from those at longer experiment times due to the greater effect of convection created by the release of oil droplets that orient the fluid and particle toward the interface during initial adsorption. Despite that, to gain insight about the timescale and kinetics of LNP adsorption, the initial interfacial tension was used herein in calculations. The percentage of initial interfacial tension at different time points, *i.e*., 1, 10, or 60 min, was calculated according to Equation 1.

(1) $$IFT_{\%}=\frac{IFT_{t}}{IFT_{i}}\;\;\times 100$$

where *IFT_%._* is the percentage of initial interfacial tension (%), *IFT_t_* is the interfacial tension obtained as the average value of the 10 first frames recorded after reaching the target times of 1 and 10 min, or the final 20 frames recorded after reaching the 60 min of measurement (mN/m), and *IFT_i_* is the initial interfacial tension obtained as the average value of the 10 first frames recorded (mN/m).

The kinetics of LNP adsorption at the oil‐water interface at the beginning of the experiments was assessed based on the constant rate of interfacial tension decrease obtained according to Equation 2. The time range of 20 s was defined for the calculation of the constant rate of interfacial tension decrease after plotting the natural logarithm of normalized interfacial tension values against the experimental times.

(2) $$IFT_{rate}=\frac{IFT_{i}\;-IFT_{t}}{t}$$

where *IFT_rate_* is the constant rate of interfacial tension decrease (mN/m s^−1^), *IFT_i_* is the first interfacial tension value obtained at the beginning of the experiment (mN/m), *IFT_t_* is the interfacial tension at 20 s (mN/m), and *t* is 20 s.

Five parameters were investigated for their effect on the adsorption of LNPs at the interface of oil–water systems: 1) type of LNPs, namely BB‐, LB‐, and PB‐LNPs; 2) type of oil‐water system using hexadecane‐water or rapeseed oil‐water systems; 3) pH; 4) LNP concentration; and 5) ionic strength. Studying the pH, the LNP dispersions were diluted to 0.07 mg mL^−1^ using 25 mM citric acid solutions at pH 3, 5, 7, and 9 (adjusted with 0.5 m HCl or 10 m NaOH, accordingly). Studying the LNP concentration, LNP dispersions were diluted using 25 mM citric acid solution at pH 5 to the final concentrations of 0.01, 0.04, 0.07, and 0.10 mg mL^−1^. Studying the ionic strength, LNP dispersions were diluted to 0.07 mg mL^−1^ using 25, 100, 175, and 250 mM citric acid solutions, all adjusted to pH 5. To avoid oil droplet expansion during experiments due to the water diffusion, hexadecane, and rapeseed oil were saturated in water prior to measurements.

### Case Study

Application of BB‐LNPs and BB industrial lignin as a stabilizer for rapeseed oil‐in‐water emulsion – methodology and characterization: The BB‐LNPs were chosen, among the LNPs herein investigated, to the emulsion preparation case study due to their chemical composition, industrial availability, and adsorption performance in diverse oil‐water systems, as confirmed by the interfacial tension study. The emulsion stabilization capacity of BB‐LNPs was investigated as a function of pH. Four 0.15% (w/w) BB‐LNP dispersions were prepared at pH 2.0, 3.6, 5.0, and 8.0. Dispersion at pH 3.6 was achieved by the simple dilution of stock BB‐LNP dispersion in water to the target concentration. Other pH conditions were adjusted using 0.5 m HCl or 0.5 m NaOH solutions. The BB‐LNP dispersions were then supplemented with 5% (w/w) rapeseed oil to the total mass of 70 g and mixed using an Ultra‐Turrax (T‐18 basic, IKA, Staufen, Germany) equipped with a disperser‐type stirrer at 22 000 rpm for 2 min to form a coarse emulsion. Then, the coarse emulsion was subjected to a microfluidizer (Microfluidizer 110Y, Microfluidics, Westwood, MA, USA) configured with 75 µm (Y‐type F20Y) and 200 µm (Z‐type H30Z) chambers in a series at a pressure of 800 bar for a total of four passes. Emulsion was collected during the fifth pass aiming to obtain finer droplet size. Alternatively, a sample from the BB‐LNP emulsion prepared at pH 8.0 was readjusted to pH 5.0 and its stability was investigated. BB industrial lignin was also used as a stabilizer for emulsification at pH 8.0 with subsequent pH readjustment to pH 5.0 following the same conditions used for emulsion preparation from LNPs, i.e, 0.15% (w/w) lignin, 5% (w/w) rapeseed oil, 800 bar pressure in microfluidizer, and four passes. Similarly, a control emulsion was prepared in the absence of a stabilizer at pH 8.0 and a sample from it was also readjusted to pH 5.0 after emulsification.

The stability of emulsions was monitored using Turbiscan Lab Expert (Formulation, Toulouse, France) at a wavelength of 800 nm. Measurements from transmitted and backscattering light intensities were combined for assessing the TSI of the emulsions using the Turbisoft version 1.2 software (Formulation, Toulouse, France). The first measurement was performed just after emulsion preparation at day 0 and the following measurements were performed at several time points: over 22 days for the emulsions prepared from BB‐LNPs, 17 days for the emulsion prepared from BB industrial lignin, and 16 days for control emulsions. Emulsions were stored at room temperature during the storage stability study.

The morphology of the emulsions stabilized by BB‐LNPs was investigated using an optical light microscope (Axiolab, Carl Zeiss Inc., Oberkochen, Germany) immediately after emulsion preparation by observing an emulsion drop on a glass slide.

## Conflict of Interest

The authors declare no conflict of interest.

## Supporting information



Supporting Information

## Data Availability

The data that support the findings of this study are available in the supplementary material of this article.
