# Supplementary material for: Modulating the Spontaneous Adsorption of Lignin Nanoparticle at Oil‐Water Interfaces
Source: Macromol Rapid Commun. 2025 May 14;46(23):2500120. doi: 10.1002/marc.202500120 (PMC12687680; doi:10.1002/marc.202500120)
Supplement: Supplementary file 1 — Supporting Information [file MARC-46-2500120-s001.pdf]

Supporting Information

Modulating the spontaneous adsorption of lignin nanoparticle at oil-water interfaces

Danila M. de Carvalho, Maarit H. Lahtinen, Patrícia Figueiredo, Sami P. Hirvonen, Sami Hietala, and Kirsi S. Mikkonen**

Results and Discussion

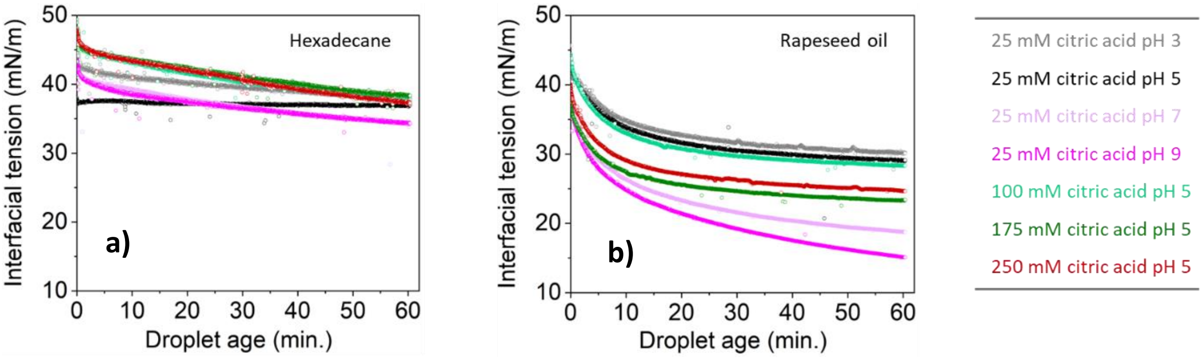

Figure S1. Control curves for the interfacial tension between the various citric acid solutions and a) hexadecane and b) rapeseed oil monitored for 60 minutes.

Table S1. Interfacial tension (IFT) between hexadecane-water and rapeseed oil-water controls showing rate of IFT decrease.

Water phase	Hexadecane-water		Rapeseed oil-water	
	IFT[a)] [mN/m]	Rate of IFT decrease[b)] [mN/m/min]	IFT[a)] [mN/m]	Rate of IFT decrease[b)] [mN/m/min]
25 mM citric acid pH 3	37.8	0.102	30.2	0.215
25 mM citric acid pH 5	36.9	0.015	29.0	0.245
25 mM citric acid pH 7	34.2	0.147	18.7	0.324
25 mM citric acid pH 9	34.4	0.162	15.1	0.390
100 mM citric acid pH 5	37.6	0.155	28.3	0.263
175 mM citric acid pH 5	38.3	0.148	23.2	0.234
250 mM citric acid pH 5	37.3	0.176	24.7	0.254

[a)]Interfacial tension at 60 minutes. [b)]Rate of interfacial tension decrease calculated based on the total duration of experiments, *i.e.*, 60 minutes. Although the rate of IFT decrease was not constant along the whole duration of the experiments (see the profile of the curves in Figure S1), the values provided quantitative information on how the IFT decreased during experiments in control systems.

Table S2. Percentage of the initial interfacial tension (%) observed at 1, 10, and 60 minutes of measurement in hexadecane-water interface. The LNP dispersion used herein varied in LNP concentration (0.01-0.10 mg/mL), pH (3-9), and ionic strength (25-250 mM citric acid). Samples highlighted in bold were assessed at the fixed conditions of 0.07 mg/mL LNPs, pH 5, and 25 mM citric acid, when studying the different variables.

LNPs	Droplet age, min.	25 mM citric acid, pH 5				25 mM citric acid, 0.07 mg/mL LNPs				pH 5, 0.07 mg/mL LNPs			
		0.01 mg/mL	0.04 mg/mL	**0.07 mg/mL**	0.10 mg/mL	pH 3	**pH 5**	pH 7	pH 9	**25 mM**	100 mM	175 mM	250 mM
BB	1	93.2	85.2	**84.5**	83.1	85.7	**84.5**	81.2	81.4	**84.5**	82.1	85.4	83.0
	10	78.8	70.9	**72.2**	69.5	74.7	**72.2**	66.8	67.0	**72.2**	68.0	71.7	69.4
	60	64.2	61.8	**64.3**	61.3	67.0	**64.3**	58.1	57.2	**64.3**	64.7	64.9	60.7
LB	1	96.2	92.8	**91.7**	89.3	93.8	**91.7**	88.4	79.6	**91.7**	86.7	89.0	88.7
	10	91.9	82.6	**80.8**	78.3	85.4	**80.8**	72.6	62.3	**80.8**	72.0	76.3	74.2
	60	83.9	73.3	**71.6**	69.5	77.1	**71.6**	61.2	51.8	**71.6**	63.5	67.1	65.7
PB	1	92.6	87.9	**84.9**	81.0	88.5	**84.9**	80.0	83.3	**84.9**	84.0	83.9	83.4
	10	81.0	72.2	**71.1**	68.2	74.2	**71.1**	66.5	70.9	**71.1**	71.1	69.6	69.6
	60	67.4	62.4	**61.5**	59.4	65.3	**61.5**	57.9	61.6	**61.5**	62.9	61.5	61.6

Table S3. Percentage of the initial interfacial tension (%) observed at 1, 10, and 60 minutes of measurement in rapeseed oil-water interface. The LNP dispersion used herein varied in LNP concentration (0.01-0.10 mg/mL), pH (3-9), and ionic strength (25-250 mM citric acid). Samples highlighted in bold were assessed at the fixed conditions of 0.07 mg/mL LNPs, pH 5, and 25 mM citric acid, when studying the different variables.

LNPs	Droplet age, min.	25 mM citric acid, pH 5				25 mM citric acid, 0.07 mg/mL LNPs				pH 5, 0.07 mg/mL LNPs			
		0.01 mg/mL	0.04 mg/mL	**0.07 mg/mL**	0.10 mg/mL	pH 3	**pH 5**	pH 7	pH 9	**25 mM**	100 mM	175 mM	250 mM
BB	1	90.8	89.0	**85.4**	86.2	91.4	**85.4**	87.0	79.6	**85.4**	85.6	84.0	84.6
	10	73.4	73.3	**70.1**	72.1	77.0	**70.1**	62.8	59.2	**70.1**	72.8	68.2	69.1
	60	64.2	66.3	**62.0**	64.8	68.8	**62.0**	48.4	42.0	**62.0**	62.2	59.6	60.7
LB	1	91.1	88.9	**91.9**	88.0	90.2	**91.9**	82.8	91.9	**91.9**	88.3	88.4	89.8
	10	71.4	68.8	**75.3**	69.7	73.9	**75.3**	61.1	69.8	**75.3**	68.3	67.7	69.6
	60	62.0	59.7	**66.4**	60.8	65.6	**66.4**	46.2	47.7	**66.4**	58.1	57.4	59.0
PB	1	90.7	87.4	**85.4**	85.4	89.4	**85.4**	81.8	78.8	**85.4**	84.8	88.7	88.8
	10	73.4	70.9	**70.9**	71.6	71.9	**70.9**	63.9	57.5	**70.9**	67.9	70.9	70.0
	60	64.2	62.3	**62.7**	64.0	63.6	**62.7**	51.9	40.3	**62.7**	58.9	61.4	60.2

No[a)]	BB-LNP	LB-LNP	PB-LNP
1	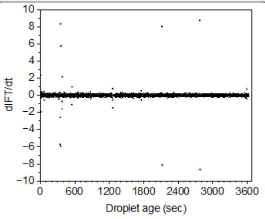	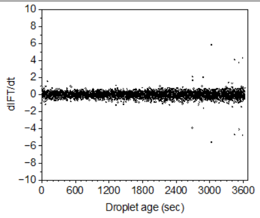	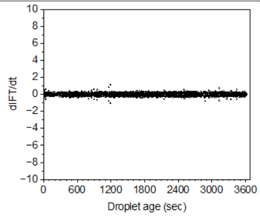
2/7/9	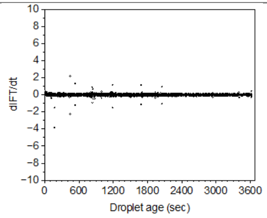	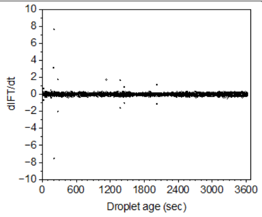	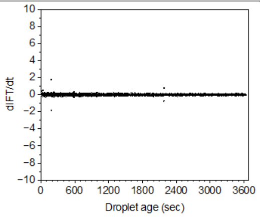
3	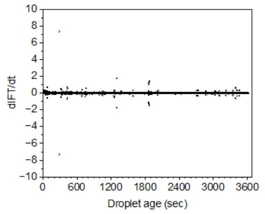	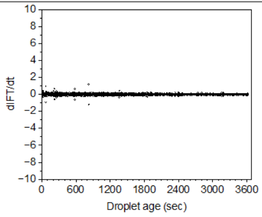	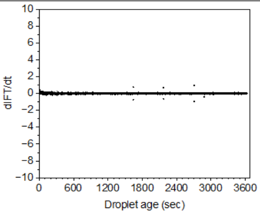
4	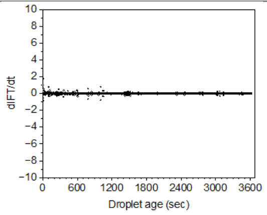	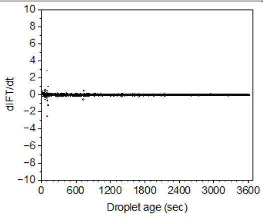	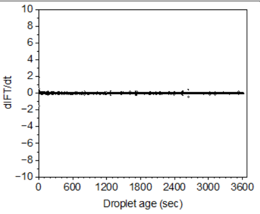
5	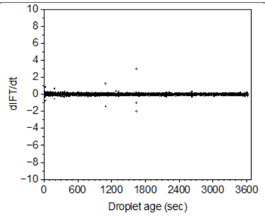	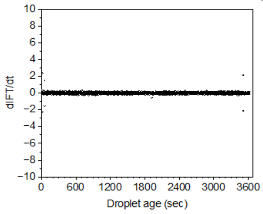	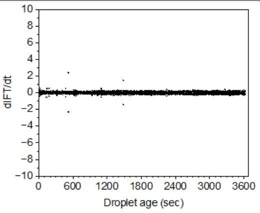
6	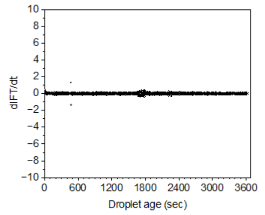	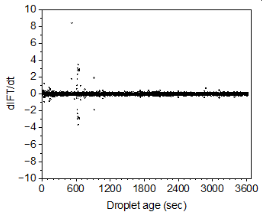	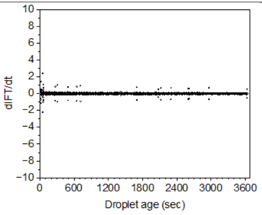
8	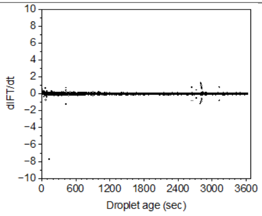	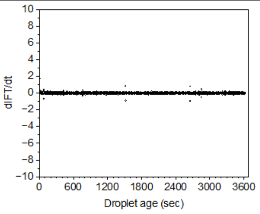	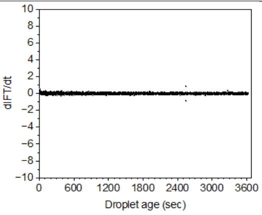
10	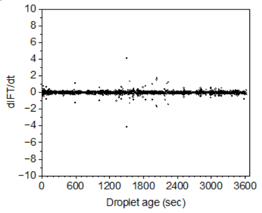	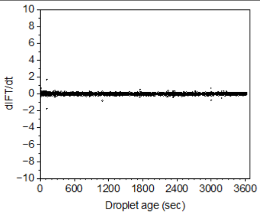	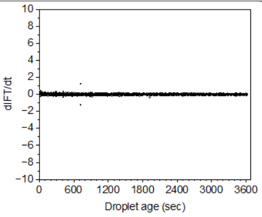

11

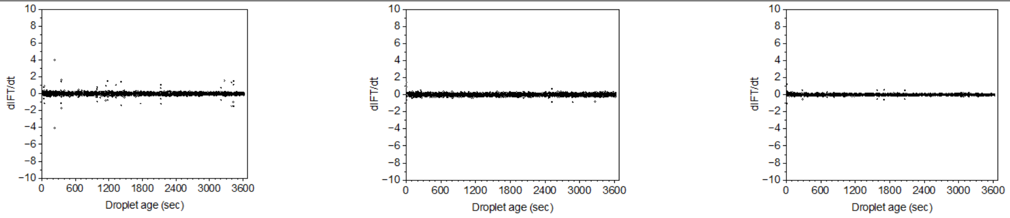

12

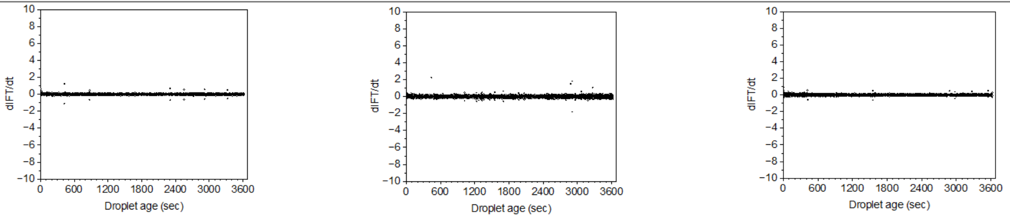

Figure S2. Rate of interfacial tension change expressed as dIFT/dt. a)Conditions used for interfacial tension measurements: 1: 0.07 mg/mL LNPs, 25 mM citric acid at pH 3; 2/7/9: 0.07 mg/mL LNPs, 25 mM citric acid at pH 5; 3: 0.07 mg/mL LNPs, 25 mM citric acid at pH 7; 4: 0.07 mg/mL LNPs, 25 mM citric acid at pH 9; 5: 0.01 mg/mL LNPs, 25 mM citric acid at pH 5; 6: 0.04 mg/mL LNPs, 25 mM citric acid at pH 5; 8: 0.10 mg/mL LNPs, 25 mM citric acid at pH 5; 10: 0.07 mg/mL LNPs, 100 mM citric acid at pH 5; 11: 0.07 mg/mL LNPs, 175 mM citric acid at pH 5; and 12: 0.07 mg/mL LNPs, 250 mM citric acid at pH 5.

Table S4. Zeta potential of LNPs in different pH conditions.

Dispersed phase	Zeta potential [mV]			Source
	BB-LNP	LB-LNP	PB-LNP	
25 mM CA pH 3	-18	-14	-17	Figueiredo et al.[1]
25 mM CA pH 5	-34	-21	-34	Figueiredo et al.[1]
25 mM CA pH 7	-45	-26	-46	Figueiredo et al.[1]
25 mM CA pH 9	-25	-19	-25	This study[a)]

Note: CA denotes citric acid solution. a)Condition for zeta-potential determination followed that described by Figueiredo et al.[1]

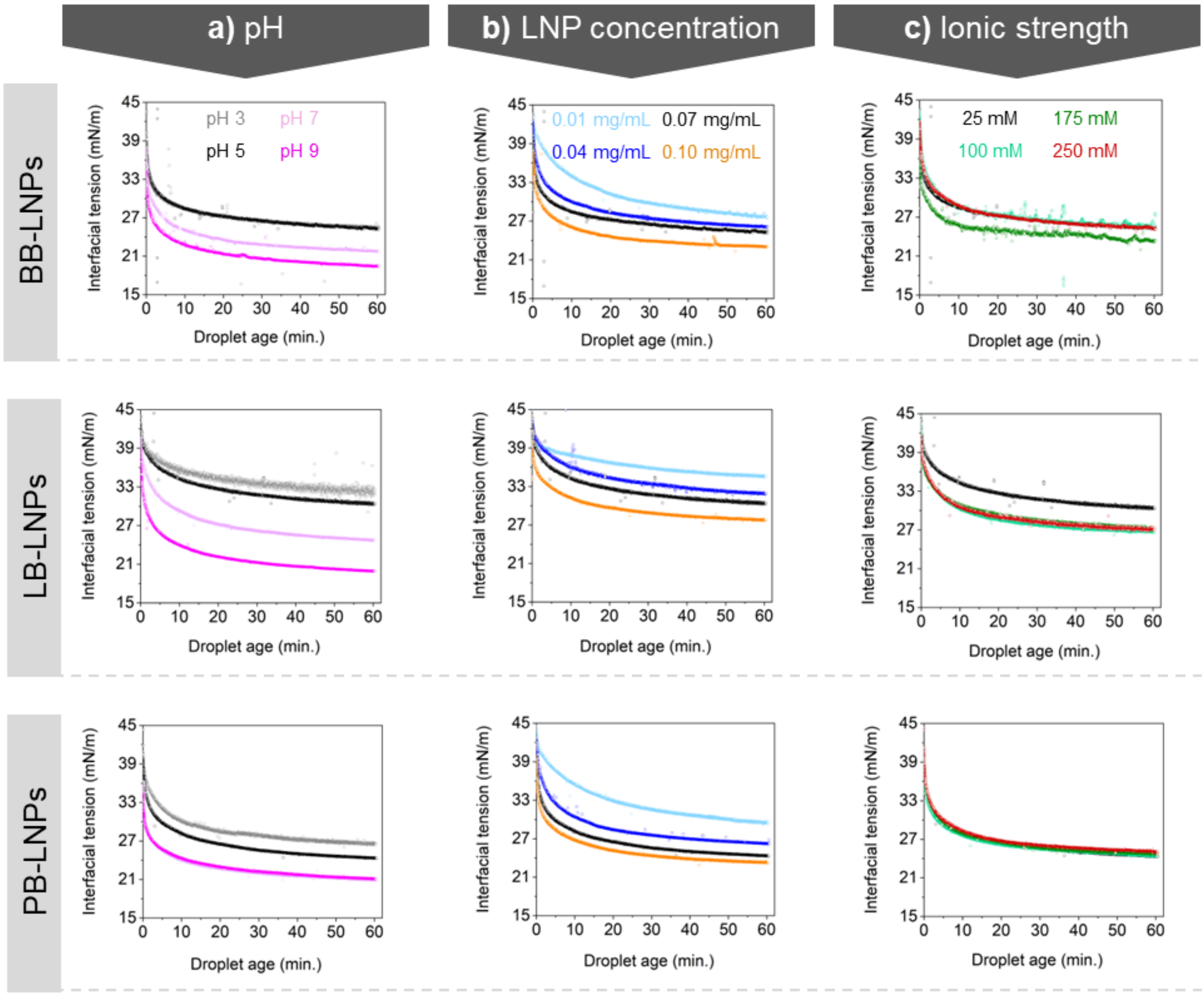

Figure S3. Curves of interfacial tension of LNP dispersion-hexadecane as a function of time for various conditions of a) pH, b) LNP concentration, and c) ionic strength. Unless otherwise specified, conditions for pH, LNPs concentration, and ionic strength were fixed at 5, 0.07 mg/mL, and 25 mM citric acid, respectively. Studying the different variables, the measurement assessed at the fixed condition is represented as black curves.

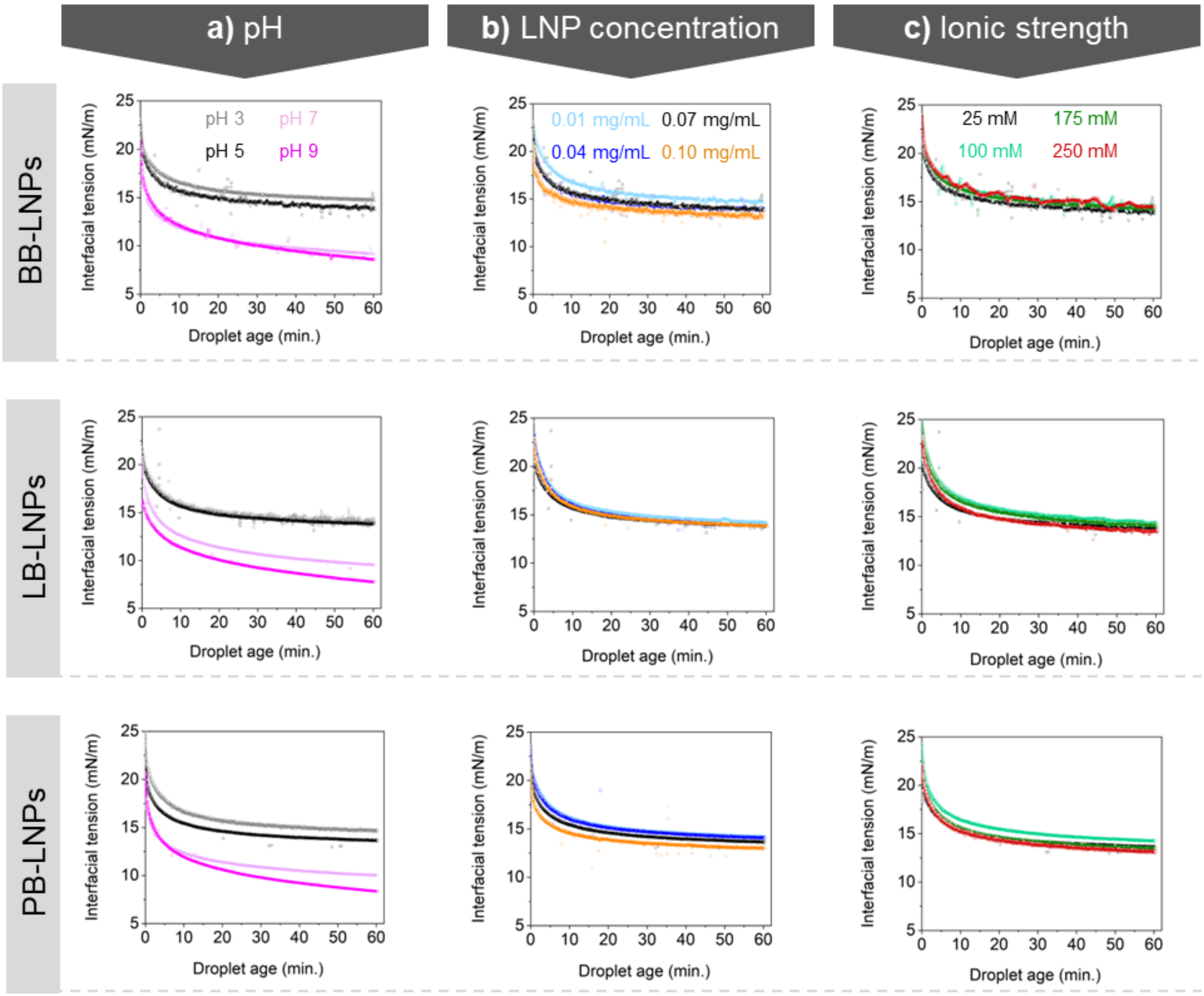

Figure S4. Curves of interfacial tension of LNP dispersion-rapeseed oil as a function of time for various conditions of a) pH, b) LNP concentration, and c) ionic strength. Unless otherwise specified, conditions for pH, LNPs concentration, and ionic strength were fixed at 5, 0.07 mg/mL, and 25 mM citric acid, respectively. Studying the different variables, the measurement assessed at the fixed condition is represented as black curves.

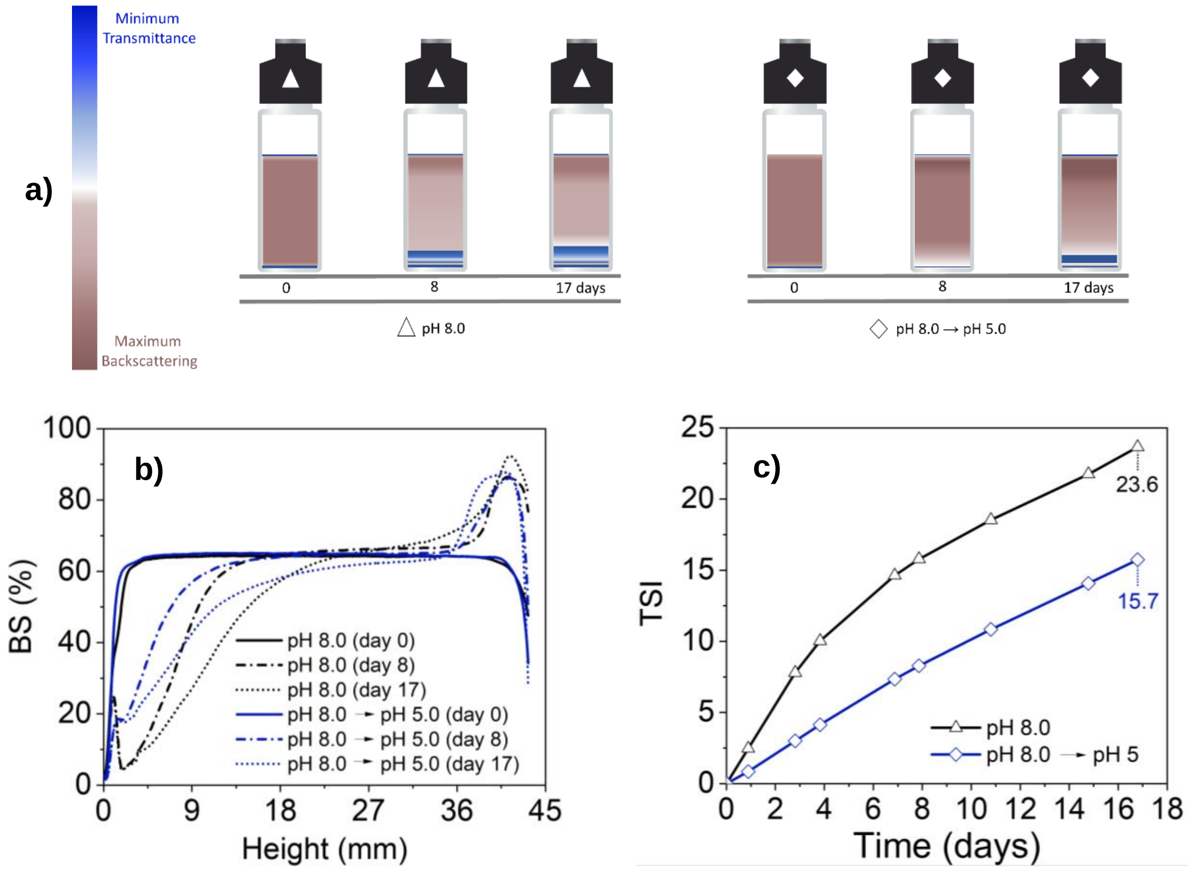

Figure S5. a) Profile of emulsions stabilized by BB technical lignin prepared at pH 8.0 and prepared at pH 8.0 and readjusted to pH 5.0 based on the transmittance and backscattering intensities. b) Backscattering intensity of emulsions prepared at pH 8.0 and prepared at pH 8.0 and readjusted to pH 5.0 assessed at days 0, 8, and 17 and c) global Turbiscan Stability Index (TSI) of emulsions prepared at pH 8.0 and prepared at pH 8.0 and readjusted to pH 5.0 monitored over 17 days of storage. For the storage period of 17 days, the TSI of emulsions prepared using BB-LNPs at pH 8 (12.8) and prepared at pH 8.0 and readjusted to pH 5.0 (11.8) were substantially lower that those observed for emulsions stabilized by BB technical lignin.

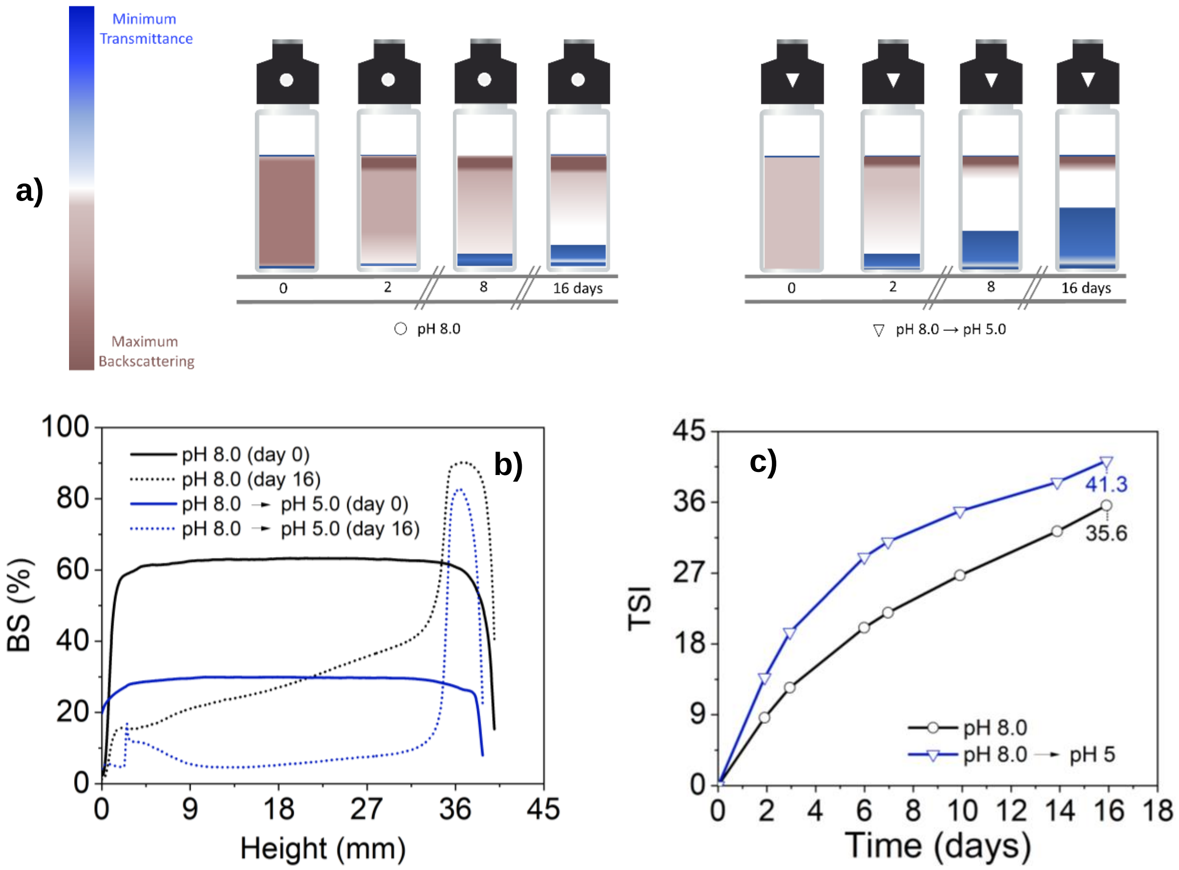

Figure S6. a) Profile of control emulsions (no stabilizer used) at pH 8.0 and prepared at pH 8.0 and readjusted to pH 5.0 based on the transmittance and backscattering intensities. b) Backscattering intensity of control emulsions prepared at pH 8.0 and prepared at pH 8.0 and readjusted to pH 5.0 assessed at days 0 and 16 and c) global TSI of control emulsions prepared at pH 8.0 and prepared at pH 8.0 and readjusted to pH 5.0 monitored over 16 days of storage. In the absence of stabilizers, even the gentle magnetic stirring applied for pH adjustment was likely enough to initiate the process for phase separation of rapeseed oil-in-water emulsion, as confirmed by the higher TSI value obtained for emulsion prepared at pH 8 and readjusted to pH 5 after emulsification compared to that in which no readjustment of pH was performed.

Experimental Section

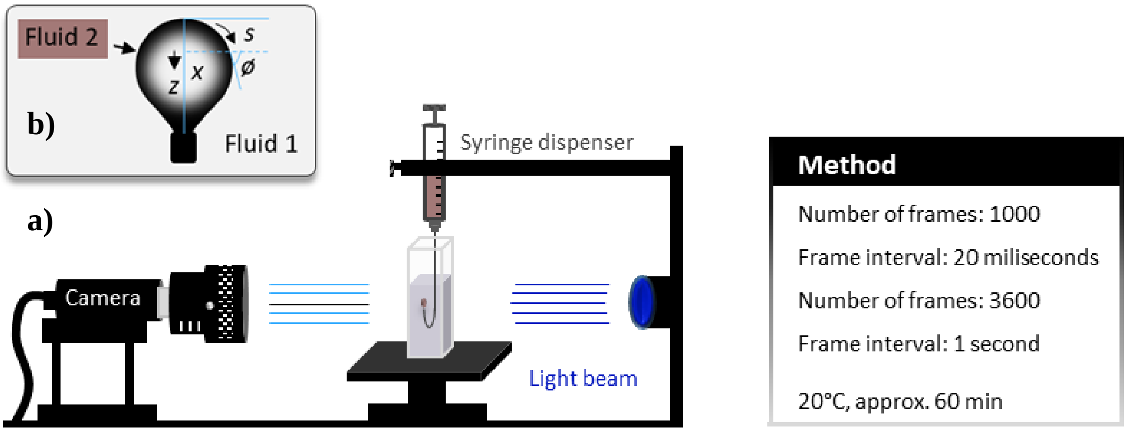

Figure S7. Simplified representation of a) method used for the optical tensiometry analysis performed to assess the interface of oil and BB-, LB-, and PB-LNP dispersions and b) the profile of the image recorded containing the shadow of the drop of oil (Fluid 2) surrounded by the LNPs dispersions (Fluid 1) in which LNPs adsorbed at the fluid-fluid interface and contributed to decreasing the interfacial tension. The Young-Laplace fitting (Equation S1) was applied to calculate the interfacial tension. In calculation, the values of 0.7714 g/cm^3 and 0.9091 g/cm^3 were used for the density of hexadecane and rapeseed oil, respectively. The estimation of the drop profile was calculated using the Equation S2, Equation S3, and Equation S4 and is expressed in a differential dimensionless form.

$$\gamma = \frac{\Delta\rho g \times R_0^2}{\beta} \tag{S1}$$

$$\frac{d\text{ø}}{ds} = 2 + \beta z - \frac{\sin \text{ø}}{x} \tag{S2}$$

$$\frac{dx}{ds} = \cos \text{ø} \tag{S3}$$

$$\frac{dz}{ds} = \sin \text{ø} \tag{S4}$$

where γ is the interfacial tension (mN/m), $\Delta\rho$ is the density difference between Fluid 1 and Fluid 2, g is the gravitational constant (negative for pendant-drops), R_0 is the radius of the drop curvature at the apex, β is the shape factor calculated from the Cartesian coordinates x and z, and s is to the contour length along the pendant-drop determined from the drop apex.

Table S5. Average volume (µL) of hexadecane and rapeseed oil released as the pendant-drop in control dispersions and monitored for 60 minutes.

Dispersed phase	Hexadecane	Rapeseed oil
25 mM CA pH 3	7.29 ± 0.03	7.79 ± 0.02
25 mM CA pH 5	7.59 ± 0.07	7.79 ± 0.05
25 mM CA pH 7	7.80 ± 0.03	7.41 ± 0.07
25 mM CA pH 9	7.58 ± 0.01	8.38 ± 0.07
100 mM CA pH 5	7.62 ± 0.02	7.57 ± 0.03
175 mM CA pH 5	7.49 ± 0.03	7.28 ± 0.03
250 mM CA pH 5	7.70 ± 0.01	7.71 ± 0.01

Note: CA denotes citric acid solution.

Table S6. Average volume (µL) of hexadecane and rapeseed oil released as the pendant-drop in LNP dispersions and monitored for 60 minutes.

No[a)]	Hexadecane			Rapeseed oil		
	BB	LB	PB	BB	LB	PB
1	7.99 ± 0.16	7.73 ± 0.09	7.84 ± 0.07	8.44 ± 0.01	7.97 ± 0.31	8.29 ± 0.02
2/7/9	8.21 ± 0.09	7.59 ± 0.04	7.68 ± 0.09	8.25 ± 0.01	7.77 ± 0.01	8.42 ± 0.01
3	8.18 ± 0.17	7.94 ± 0.06	7.48 ± 0.03	7.93 ± 0.02	7.85 ± 0.06	7.92 ± 0.02
4	7.96 ± 0.01	7.41 ± 0.18	7.92 ± 0.03	8.13 ± 0.01	7.68 ± 0.06	8.29 ± 0.01
5	7.75 ± 0.09	7.63 ± 0.04	8.04 ± 0.06	7.98 ± 0.00	8.16 ± 0.01	7.76 ± 0.02
6	8.12 ± 0.08	7.88 ± 0.05	7.26 ± 0.08	8.09 ± 0.01	7.66 ± 0.17	8.38 ± 0.02
8	7.75 ± 0.05	7.90 ± 0.03	7.80 ± 0.10	7.99 ± 0.01	8.19 ± 0.01	7.75 ± 0.04
10	7.92 ± 0.23	7.51 ± 0.01	7.94 ± 0.01	8.29 ± 0.03	8.09 ± 0.01	7.77 ± 0.00
11	7.69 ± 0.02	7.78 ± 0.01	7.90 ± 0.01	8.25 ± 0.03	7.77 ± 0.01	7.55 ± 0.01
12	7.68 ± 0.04	7.41 ± 0.02	7.67 ± 0.01	7.54 ± 0.01	7.70 ± 0.03	7.61 ± 0.02

[a)]Conditions used for interfacial tension measurements: 1: 0.07 mg/mL LNPs, 25 mM citric acid at pH 3; 2/7/9: 0.07 mg/mL LNPs, 25 mM citric acid at pH 5; 3: 0.07 mg/mL LNPs, 25 mM citric acid at pH 7; 4: 0.07 mg/mL LNPs, 25 mM citric acid at pH 9; 5: 0.01 mg/mL LNPs, 25 mM citric acid at pH 5; 6: 0.04 mg/mL LNPs, 25 mM citric acid at pH 5; 8: 0.10 mg/mL LNPs, 25 mM citric acid at pH 5; 10: 0.07 mg/mL LNPs, 100 mM citric acid at pH 5; 11: 0.07 mg/mL LNPs, 175 mM citric acid at pH 5; and 12: 0.07 mg/mL LNPs, 250 mM citric acid at pH 5.

Table S7. Average of the area (mm^2) of hexadecane and rapeseed oil released as the pendant-drop in control dispersions and monitored for 60 minutes.

Dispersed phase	Hexadecane	Rapeseed oil
25 mM CA pH 3	18.1 ± 0.1	18.6 ± 0.0
25 mM CA pH 5	18.4 ± 0.1	18.7 ± 0.1
25 mM CA pH 7	19.0 ± 0.0	18.0 ± 0.0
25 mM CA pH 9	18.6 ± 0.0	19.7 ± 0.1
100 mM CA pH 5	18.7 ± 0.0	18.3 ± 0.0
175 mM CA pH 5	18.5 ± 0.0	17.9 ± 0.0
250 mM CA pH 5	18.7 ± 0.0	18.5 ± 0.0

Note: CA denotes citric acid solution.

Table S8. Average of the area (mm^2) of hexadecane and rapeseed oil released as the pendant-drop in LNP dispersions and monitored for 60 minutes.

No[a)]	Hexadecane			Rapeseed oil		
	BB	LB	PB	BB	LB	PB
1	19.3 ± 0.2	18.9 ± 0.1	19.1 ± 0.1	20.0 ± 0.0	19.2 ± 0.5	19.8 ± 0.0
2/7/9	19.7 ± 0.2	18.6 ± 0.1	18.8 ± 0.2	19.7 ± 0.0	18.9 ± 0.0	20.0 ± 0.0
3	19.6 ± 0.2	19.3 ± 0.1	18.5 ± 0.0	19.2 ± 0.0	19.1 ± 0.1	19.2 ± 0.0
4	19.3 ± 0.0	18.4 ± 0.3	19.2 ± 0.0	19.5 ± 0.0	18.8 ± 0.1	19.8 ± 0.0
5	18.9 ± 0.2	18.7 ± 0.1	19.4 ± 0.1	19.3 ± 0.0	19.6 ± 0.0	18.9 ± 0.0
6	19.5 ± 0.1	19.1 ± 0.1	18.1 ± 0.1	19.4 ± 0.0	18.7 ± 0.3	19.9 ± 0.0
8	18.9 ± 0.1	19.1 ± 0.0	19.0 ± 0.1	19.3 ± 0.0	19.6 ± 0.0	18.9 ± 0.1
10	19.2 ± 0.3	18.5 ± 0.0	19.2 ± 0.0	19.8 ± 0.0	19.4 ± 0.0	18.9 ± 0.0
11	18.8 ± 0.0	19.0 ± 0.0	19.2 ± 0.0	19.7 ± 0.0	19.0 ± 0.0	18.6 ± 0.0
12	18.8 ± 0.1	18.4 ± 0.0	18.8 ± 0.0	18.6 ± 0.0	18.8 ± 0.0	18.7 ± 0.0

[a)]Conditions used for interfacial tension measurements: 1: 0.07 mg/mL LNPs, 25 mM citric acid at pH 3; 2/7/9: 0.07 mg/mL LNPs, 25 mM citric acid at pH 5; 3: 0.07 mg/mL LNPs, 25 mM citric acid at pH 7; 4: 0.07 mg/mL LNPs, 25 mM citric acid at pH 9; 5: 0.01 mg/mL LNPs, 25 mM citric acid at pH 5; 6: 0.04 mg/mL LNPs, 25 mM citric acid at pH 5; 8: 0.10 mg/mL LNPs, 25 mM citric acid at pH 5; 10: 0.07 mg/mL LNPs, 100 mM citric acid at pH 5; 11: 0.07 mg/mL LNPs, 175 mM citric acid at pH 5; and 12: 0.07 mg/mL LNPs, 250 mM citric acid at pH 5.

References

[1] P. Figueiredo, M. H. Lahtinen, M. B. Agustin, D. M. de Carvalho, S.-P. Hirvonen, P. A. Penttilä, K. S. Mikkonen, *ChemSusChem* **2021**, *14*, 1-14.
